# Supplementary material for: A model framework for projecting the prevalence and impact of Long-COVID in the UK
Source: PLoS One. 2021 Dec 2;16(12):e0260843. doi: 10.1371/journal.pone.0260843 (PMC8639065; doi:10.1371/journal.pone.0260843)
Supplement: Attachment — Submitted filename: PlosOneRebuttalLetter_20210813.docx [file pone.0260843.s001.docx]

Dr Chris Martin Managing Director,
Crystallise Ltd 19 Saffron Court,
Southfields Business Park,
Basildon,
Essex
SS15 6SS

The Editor

PLOS One

Dear Dr Chen,

This is the rebuttal letter for the resubmission of the paper “A model framework for projecting the prevalence and impact of Long-COVID in the UK”.

For clarity, we reproduce the feedback given in bold italics, and then give our response in black type. Where possible, we have included the sentences or paragraphs added or edited to address the reviewer concerns.

You asked for the following in our resubmission:

***	***A rebuttal letter that responds to each point raised by the academic editor and reviewer(s). You should upload this letter as a separate file labeled 'Response to Reviewers'.***

This is this current document.

****	A marked-up copy of your manuscript that highlights changes made to the original version. You should upload this as a separate file labeled 'Revised Manuscript with Track Changes'.***

This will be uploaded as a separate document.

****	An unmarked version of your revised paper without tracked changes. You should upload this as a separate file labeled 'Manuscript'.***

This will be uploaded as a separate document.

***If you would like to make changes to your financial disclosure, please include your updated statement in your cover letter. Guidelines for resubmitting your figure files are available below the reviewer comments at the end of this letter.***

The revised cover letter will be uploaded as a separate document.

Figure files have been updated in the “Revised Manuscript with Track Changes” file and will also be uploaded as separate TIFF image files.

***If applicable, we recommend that you deposit your laboratory protocols in protocols.io to enhance the reproducibility of your results. Protocols.io assigns your protocol its own identifier (DOI) so that it can be cited independently in the future.***

***Additionally, PLOS ONE offers an option for publishing peer-reviewed Lab Protocol articles, which describe protocols hosted on protocols.io.***

This is not applicable as no laboratory protocols were used.

***Journal Requirements:***

***When submitting your revision, we need you to address these additional requirements.***

***1. Please ensure that your manuscript meets PLOS ONE's style requirements, including those for file naming. The PLOS ONE style templates can be found at***

We have worked through the style guides for Manuscript, Figures, Tables, and References and believe that the manuscript now meets the style requirements.

2. ***We note that longCOVID/LICENSE data presented as the supporting information on GritHub in your submission contain copyrighted images/data. All PLOS content is published under the Creative Commons Attribution License (CC BY 4.0), which means that the manuscript, images, and Supporting Information files will be freely available online, and any third party is permitted to access, download, copy, distribute, and use these materials in any way, even commercially, with proper attribution. For more information, see our copyright guidelines:***

***We require you to either (1) present written permission from the copyright holder to publish these figures specifically under the CC BY 4.0 license, or (2) remove the figures from your submission:***

***2.1. You may seek permission from the original copyright holder of Figure/Data to publish the content specifically under the CC BY 4.0 license.***

The screenshot reproductions of figures from Haplin et al and Garrigues et al have been removed from “Utility” sheet in the LongCOVID_ModelDevelopment_v13.xlsx file in the Github repository. Note that the new version is now called version 14 rather than version 13.

3. ***We note that you have indicated that data from this study are available upon request. PLOS only allows data to be available upon request if there are legal or ethical restrictions on sharing data publicly. For information on unacceptable data access restrictions, …***

***In your revised cover letter, please address the following prompts:***

***a) If there are ethical or legal restrictions on sharing a de-identified data set, please explain them in detail (e.g., data contain potentially identifying or sensitive patient information) and who has imposed them (e.g., an ethics committee). Please also provide contact information for a data access committee, ethics committee, or other institutional body to which data requests may be sent.***

b***) If there are no restrictions, please upload the minimal anonymized data set necessary to replicate your study findings as either Supporting Information files or to a stable, public repository and provide us with the relevant URLs, DOIs, or accession numbers.***

***We will update your Data Availability statement on your behalf to reflect the information you provide.***

This will be addressed in the online submission portal. The response to the “Data Availability” question should have been “Yes – all data are fully available without restriction” and that is what appears during resubmission. For the avoidance of doubt, there are no restrictions of any kind on the sharing of the data.

***4. Please amend either the title on the online submission form (via Edit Submission) or the title in the manuscript so that they are identical.***

It would appear that the title in the submission and the manuscript are identical already.

***FUNDING and Conflict of Interest***

***1. Thank you for stating the following in the Competing Interests section:***

***"NO authors have competing interests"***

***We note that one or more of the authors are employed by a commercial company: Lloyds Banking Group, Hannover Re, Crystallise Ltd.***

***1.1. Please provide an amended Funding Statement declaring this commercial affiliation, as well as a statement regarding the Role of Funders in your study. If the funding organization did not play a role in the study design, data collection and analysis, decision to publish, or preparation of the manuscript and only provided financial support in the form of authors' salaries and/or research materials, please review your statements relating to the author contributions, and ensure you have specifically and accurately indicated the role(s) that these authors had in your study. You can update author roles in the Author Contributions section of the online submission form.***

***Please also include the following statement within your amended Funding Statement.***

***"The funder provided support in the form of salaries for authors [insert relevant initials], but did not have any additional role in the study design, data collection and analysis, decision to publish, or preparation of the manuscript. The specific roles of these authors are articulated in the 'author contributions' section."***

***If your commercial affiliation did play a role in your study, please state and explain this role within your updated Funding Statement.***

***1.2. Please also provide an updated Competing Interests Statement declaring this commercial affiliation along with any other relevant declarations relating to employment, consultancy, patents, products in development, or marketed products, etc.***

***Within your Competing Interests Statement, please confirm that this commercial affiliation does not alter your adherence to all PLOS ONE policies on sharing data and materials by including the following statement: "This does not alter our adherence to PLOS ONE policies on sharing data and materials."*** …

***If this adherence statement is not accurate and there are restrictions on sharing of data and/or materials, please state these. Please note that we cannot proceed with consideration of your article until this information has been declared.***

***Please include both an updated Funding Statement and Competing Interests Statement in your cover letter. We will change the online submission form on your behalf.***

***Please know it is PLOS ONE policy for corresponding authors to declare, on behalf of all authors, all potential competing interests for the purposes of transparency. PLOS defines a competing interest as anything that interferes with, or could reasonably be perceived as interfering with, the full and objective presentation, peer review, editorial decision-making, or publication of research or non-research articles submitted to one of the journals. Competing interests can be financial or non-financial, professional, or personal. Competing interests can arise in relationship to an organization or another person. …***

This will be addressed in the online submission portal and cover letter.

# **Reviewer #1:**

***Method***

**** The authors should provide the information first about the survey, study population and the study groups clearly.***

We are not certain to what this refers.

Additional information has been included on the ONS COVID symptom survey and study population:

"The national Coronavirus Infection Survey was conducted by the Office of National Statistics (ONS). This survey was carried out between April and December 2020 and included 8,193 respondents. It is considered most relevant as it contains the largest sample and provides a reference to the UK general population prevalence. The Coronavirus Infection Survey consisted of a random, a-priori, selected sample that is were invited for COVID tests and therefore would cover asymptomatic and symptomatic cases."

The methods section has been amended to be clearer as to the population and subpopulations used in our modelling process:

“Both the short-term injured and the permanently injured were divided into three mutually exclusive treatment groups: non-hospitalised, ward-based care and Intensive Treatment Unit (ITU) care (Fig 1). COVID-19-related QALY loss differed by treatment group, while the probabilities of emerging with permanent injury also varied by treatment group.”

**** Define the symptomatic COVID-19 cases and permanently injured cases***

This has been clarified:

“Total symptom prevalence at each time point was estimated as a combination of symptomatic persons with short-term injury, which decays over time, and persons with permanent injury, which has an unchanging prevalence.”

**** Define the three subgroups of symptomatic COVID-19 as well.***

This section in the introduction has been amended to make it clearer how the definition of long-covid corresponds to the three symptom phases defined by NICE:

“The National Institute for Health and Care Excellence (NICE) has defined three phases to symptoms following COVID-19 [4]. First, ‘Acute COVID-19 infection’ covers the period of active infection up to 4-weeks post-infection. Second, ‘Ongoing symptomatic COVID-19’ covers the period when infection should have ceased but persisting effects from the infection that may take time to heal may be present from 4 and 12-weeks post-infection. Third, ‘Post-COVID-19 syndrome’ is defined as ‘Signs and symptoms that develop during or following an infection consistent with COVID-19, continue for more than 12 weeks and are not explained by an alternative diagnosis.’ Long-COVID describes both ongoing symptomatic COVID-19 (the second group) as well as post-COVID-19 syndrome (the third group). Documented symptoms for long-COVID include breathlessness, fatigue, myalgia, chest pains and insomnia [5].”

**** According to recent devlepment on COVID-19 symptomatic cases, there are four types : mild, moderate, severe (hospitalized), very severe (in ICU). Why did the authors did not adopt these subgroups of symptomatic cases ?***

The four types appear to be arbitrarily defined and in any case do not appear to relate directly to the persistence of symptoms but rather the diagnosis and treatment streams. However, our manuscript does distinguish non-hospitalised, ward-based- and ICU patients as distinct groups that are both well-defined and for which some symptom persistence data was available.

**** Authors don’t define what is long COVID ?***

The introduction now references the definition of long-covid to the three symptom phase phases given by NICE:

“Long- COVID describes both ongoing symptomatic COVID-19 (the second group) as well as post-COVID-19 syndrome (the third group). Documented symptoms for long- COVID include breathlessness, fatigue, myalgia, chest pains and insomnia [5].”

**** Please add a subsection of < sensitivity analysis > performed in the method section.***

Added.

***Model :***

****In the method section, some essential information are missing and it is not easy to understand the method section without this information :***

**** It is unclear which model was used to create the framework ?***

This has hopefully now been made clearer at the start of the methods section:

“The model developed is a compartmental forecast model, and estimates QALYs lost due to COVID-19 illness, but not deaths, in the UK population of 66.6 million.”

The ‘model overview’ section gives a broad structure of the model, and then the ‘model parameters’ section goes into further detail.

Additionally, a sentence has been added to the introduction explaining the use of the term ‘framework’ in the manuscript:

“The model is presented as a framework, which can be developed as better estimates for parameterisation become available, for example for the prevalence of long-term symptoms, the QoL impact of symptoms, and the impact of vaccination programmes.”

**** The rational to choose the decay function and to keep the fixed prevalence for permanently injured group***

Again, hopefully this is now clearer:

“QALYs lost in the surviving persons were then estimated based on the prevalence of symptoms over time. Total symptom prevalence at each time point was estimated as a combination of symptomatic persons with short-term injury, which decays over time, and persons with permanent injury, which has an unchanging prevalence. Short-term symptom prevalence was modelled using a decay function based on the findings of national Coronavirus Infection Survey [9].”

**** Please add the abbrevaitions in the text such as ITU, CIS study, PTSD, etc.***

Full terms for UK, NICE, ARDS, PTSD, QOL, QALY, ONS, ITU, HRQoL, and PRO have all been added/checked. The reference to CIS study for Coronavirus Infection Survey was removed.

***Utility***

**** Please define < average utility change > ?***

Hopefully this is now clearer:

“Loss of utility due to COVID-19 was defined as the change in EQ5D score, which is a questionnaire-based measure of five well-being dimensions: mobility, self-care, usual activities, pain/discomfort, and anxiety/depression.”

Table 1 is describing the parameters used for the model and also it reported the result of the sensitivity anaysis that should be the part of the results section ?

This table does not report results of the sensitivity analysis, only the range of values used for the purpose of sensitivity analysis. We have altered the column headings to make this clearer.

***Results***

****Overall the result section seems to have insufficient information in interpretation, no table is provided.***

**** It is unclear which framework is developed ?***

Hopefully this has been addressed in the changes to the Model section:

“The model developed is a compartmental forecast model, and estimates QALYs lost due to COVID-19 illness, but not deaths, in the UK population of 66.6 million.”

**** The authors can provide a comparison of discounted and undicounted estimates and report them in a table at two time points of 1-year and 10 year time horizen.***

Done. Table 2 contains estimates for years 1-10 as a companion to the graphical representation in Figure 3.

**** In the method section, author did not report that the permanent injury from COVID-19 including lung fibrosis, the sequelae of major adverse cardiovascular events like heart attacks and strokes and psychological impacts such as PTSD, will be measured ? This information should be reported in the method section first.***

The nature of permanent injury due to COVID-19 infection and treatment is discussed in the Introduction:

“Documented symptoms for long-COVID include breathlessness, fatigue, myalgia, chest pains and insomnia [5].”

“In a study of patients with acute respiratory distress syndrome about a third of those who were previously employed were still unemployed 5-years later [7], suggesting long term disability. A dysfunctional and uncontrolled immune response can cause multi-organ damage, particularly the liver and kidneys, and disrupt the coagulation control mechanisms of the blood [8]. This can precipitate major adverse cardiovascular events which may have long-term consequences such as heart failure or hemiplegia. Data from the COVID Infection Survey study on long-COVID suggests that the risk of major adverse cardiovascular events is about ten times higher in cases with non-intensive care hospitalized patients with COVID when compared to matched controls [9]. Following treatment in critical care with acute respiratory distress syndrome, about 25% of patients have post-traumatic stress disorder (PTSD) and about 40% suffer depression [10,11].”

How this is incorporated into the model should now be clearer in the methods section:

“Total symptom prevalence at each time point was estimated as a combination of symptomatic persons with short-term injury, which decays over time, and persons with permanent injury, which has an unchanging prevalence.”

We have also removed mention of “lung fibrosis, the sequelae of major adverse cardiovascular events like heart attacks and strokes and psychological impacts such as PTSD” in the results section as these were simply used as illustrations and not explicitly measured. In hindsight, this section could confuse readers and was therefore removed.

***Discussion***

****This section is very long and less convincing.***

It is difficult to make specific changes based on this feedback. We have reduced the word count of the discussion by increasing information density. We also combined the two separate sections where symptom prevalence studies were discussed to improve the flow of the discussion.

# **Reviewer #2:**

****Regarding the methods section, I noticed the tense was often changed to the present tense. This is very confusing and I feel it is important to remain with the past tense at all times, as the methods are describing what you did. This is true as well for the description of your methods in the abstract. Therefore, "Both parts are combined..." should be changed to "Both parts were combined" on line 28.***

The model development description in the Methods section has been put into the past tense.

****This is a very minor comment, but could be of interest. You use UTI, which I assume is Intensive Treatment Unit. As this is often referred to as the ICU in the United States, it may be of interest to simply define it the first time you use this acronym (I believe on line 99).***

Done.

****In the Discussion section, on line 263 you mention, "In addition, this provides support for non-pharmaceutical interventions that reduce the transmission of the SARS-Cov-2", this is a great point. It could be of interest to provide one or several examples by finishing the sentence with, "such as..."***

Done:

“Given the socio-economic disparity in the pandemic burden, this may provide justification for spending which seeks to reduce health inequalities such as tailored public health messaging, vaccination delivery, and community access to care. In addition, this provides support for non-pharmaceutical interventions that reduce the transmission of the SARS-Cov-2, such as physical distancing and the use of face masks.”

****There appears to just be a small typo on line 267 "not return to fully to work" should be "not return fully to work"***

Done.

****This is very small, but on line 287 it should be "different types of questionnaires" with an "s" at the end***

Done.

****On line 279, I believe it should be "although NICE has published" instead of "have published"***

Done.

****The limitations section made some very interesting points. I did feel though this section could be re-organised slightly. For example, on line 307 the discussion of recall bias is very interesting but does not fit there. I would discuss recall bias under paragraph 2 where you discuss challenges with standardized measures and recording of data.***

We have made the specific change as requested.
