# Supplementary material for: A model framework for projecting the prevalence and impact of Long-COVID in the UK
Source: PLoS One. 2021 Dec 2;16(12):e0260843. doi: 10.1371/journal.pone.0260843 (PMC8639065; doi:10.1371/journal.pone.0260843)
Supplement: Attachment — Submitted filename: Response to Reviewers.docx [file pone.0260843.s002.docx]

Dr Chris Marti

Briars, Orsett Road

Horndon on the Hill

Essex

SS17 8NS

8^th^ November 2011

The Editor

Plos One

Dear Editor,

This is the rebuttal letter for the resubmission of the article: ***A model framework for projecting the prevalence and impact of Long-COVID in the UK***

**Comment 4.**

Availability of data.

The data has been freely available on the Github repository at this location:

<https://github.com/Crystallize/longCOVID>.

We gave the URL of this repository in two places in the submission, but it is not given in the body of the article itself as we assumed that this would appear as a link on the web page. To clarify this, I have added a sentence at the end of the methods section with the URL. If you do, in fact, provide the link separately, feel free to remove this sentence if you feel it appropriate.

**6. Review Comments to the Author**

Reviewer 2.

The data is available via Github from the URL given in the submission. We have added that URL to the text of the report for clarity.

Regarding the list of symptoms of long-Covid, we give details in lines 59 and 60 of the introduction.

To clear up any confusion, the authors were in no way associated with the CIS that was the main source of data of symptom prevalence and we are therefore unable to provide copies of questionnaires etc that were used. The data that we used is only that which is publicly available from the sources given in the references.

Reviewer 3.

We have removed the headings from the abstract.

We noted that there was no guidance given on headings in the abstract and that more than half of the abstracts we examined on the Plos One site had headings, so we included them. We have no problem with them being removed if that is preferred.

**Other**

I have corrected a typo in line 296 where there was a missing hyphen. Everything else is unchanged.

Thank you.

Yours sincerely,

Dr Chris Martin

Communicating author
